# Auditory Brainstem Encoding of Speech-in-Noise Predicts Word Identification, Narrative Comprehension, and Subjective Listening Effort in a Selective Attention Task

**DOI:** 10.1523/ENEURO.0272-25.2026

**Published:** 2026-09-04

**Authors:** Kelsey Mankel, Daniel C. Comstock, Brett M. Bormann, Soukhin Das, Doron Sagiv, Hilary Brodie, Lee M. Miller

**Affiliations:** ^1^School of Communication Sciences and Disorders, University of Memphis, Memphis, Tennessee 38152; ^2^Institute for Intelligent Systems, University of Memphis, Memphis, Tennessee 38111; ^3^Center for Mind and Brain, University of California, Davis, Davis, California 95618; ^4^Neuroscience Graduate Group, University of California, Davis, Davis, California 95616; ^5^Departments of Otolaryngology|Head and Neck Surgery, University of California, Davis, Davis, California 95616; ^6^Neurobiology, Physiology and Behavior, University of California, Davis, Davis, California 95616

**Keywords:** auditory brainstem response (ABR), auditory event-related potentials (ERPs), chirped speech (Cheech), electroencephalography (EEG), speech-in-noise

## Abstract

The auditory brainstem plays a crucial role in speech-in-noise (SiN) listening, refining numerous acoustic features such as pitch and spatial location under continual descending influence from the cortex. However, the difficulty of characterizing brainstem activity during continuous speech listening has obscured its functional role in ecologically valid contexts—not only the effects of selective attention on neural responses but also their impact on comprehension and listening effort. Here, we evaluated the role of the brainstem in SiN perception and other listening behaviors using a continuous, speech-based stimulus with embedded chirps (Cheech) that rapidly and effectively evoked auditory brainstem responses (ABRs) while 25 participants listened to short story narratives (*n* = 15 females, 10 males). The Cheech-modified stories were presented alone or in the presence of another spatially separated talker, and neural responses were measured throughout by EEG. Both word-level detection performance and narrative-level comprehension were evaluated, as well as subjective reports of listening effort. ABR wave V was modulated by the presence of a competing talker. Additionally, wave V peak amplitudes were associated with identification speed of the target words embedded within the stories, while faster latencies were related to better target word identification accuracy, comprehension question accuracy, and lower subjective listening effort. Collectively, our results provide evidence for the influence of brainstem encoding processes on individual speech-listening behaviors, including the ability to selectively attend to and comprehend target speech in the presence of a competing talker.

## Significance Statement

This study highlights the crucial role of the brainstem in speech-in-noise perception and higher-level language abilities like comprehension and subjective listening effort. Through the use of a novel speech stimulus blended with embedded chirps (Cheech) to evoke brainstem responses while listening to short story narratives, we show how subcortical processes relate to successful speech-listening performance across multiple behavioral metrics. These findings advance our understanding of how the brainstem supports speech perception, comprehension, and perceived effort in challenging listening environments.

## Introduction

Neural processing of speech has primarily been studied at the level of the cortex (Hickok and Poeppel, 2004). Yet, brainstem nuclei along the auditory pathway perform crucial functions relevant for processing speech such as encoding pitch and timing of spectrotemporal fluctuations (Krishnan and Gandour, 2009; Krishnan and Plack, 2011), preliminary phoneme categorization (Carter and Bidelman, 2023; Rizzi and Bidelman, 2023), and even early differentiation of competing auditory streams, likely through corticofugal modulations (Price and Bidelman, 2021). Additionally, poorer encoding of auditory brainstem responses (ABRs) has been associated with decreased speech-in-noise (SiN) performance (Papakonstantinou et al., 2011; Bramhall et al., 2015). Thus, rather than serving as a simple relay for acoustic information, the brainstem plays an active and perceptually consequential role in speech perception.

Yet, whether and how subcortical processes influence the ability to selectively attend to target sounds while filtering out background noise, as required for SiN perception, remains uncertain. ABRs were initially considered immune from effects of attention and arousal states (Jewett and Williston, 1971; Connolly et al., 1989; Gregory et al., 1989). However, midbrain responses (ABR wave V) may be modulated by selective attention of audition over another modality (Lukas, 1980; Bauer and Bayles, 1990; Kumar et al., 2023), increased cognitive load (Sörqvist et al., 2012), or cognitive interference (Brännström et al., 2020). Recently, selective attention in a dichotic listening paradigm led to enhanced ABR wave V for attended versus ignored chirps (but not tone bursts; Strauss et al., 2025). Mixed results on the influence of attention have also been demonstrated for other subcortical responses like the frequency-following response (FFR; Hoormann et al., 2000; Galbraith et al., 2003; Varghese et al., 2015), possibly due to cortical contributions (Coffey et al., 2016; cf. Bidelman, 2018). Meanwhile, regression- or correlation-based brainstem responses derived from continuous speech were more robust for attended rather than unattended talkers (Forte et al., 2017; Etard et al., 2019), and attentional modulation of these responses were related to independent tests of SiN perception (Saiz-Alía and Reichenbach, 2020).

Historically conflicting results regarding attentional modulation of brainstem activity likely reflect differences among experimental approaches, chief among them a varying degree of ecological validity. While subcortical EEG responses are best evoked by transient artificial stimuli such as isolated clicks or tone bursts, these are not always good predictors of speech perception in real-world listening environments (Skoe and Kraus, 2010; Grose et al., 2017; Smith et al., 2019; DiNino et al., 2025), nor do simple sounds adhere to naturalistic statistics known to influence midbrain encoding (Escabí et al., 2003). As a result, despite numerous studies of auditory brainstem function, its role in attentive listening and its relevance to real-world speech performance remains unclear. In order to shed light on the neural processes underlying SiN perception, a challenging, ecologically valid task using naturalistic speech with relevant outcome metrics should be used (Hamilton and Huth, 2020).

However, fundamental limitations characterize ABR recordings such as low signal-to-noise ratios (SNR), relatively small amplitudes, and the need for multiple stimulus repetitions. To address this, we have developed a technique for the rapid measurement of brainstem through cortical responses via “chirped speech” (Cheech), continuous speech stimuli blended energetically and perceptually with frequency sweeps (Backer et al., 2019; Miller et al., 2020; Shehabi et al., 2025). Our technique parallels recent efforts to derive brainstem activity during continuous speech (Forte et al., 2017; Maddox and Lee, 2018; Etard et al., 2019; Polonenko and Maddox, 2021; Kulasingham et al., 2024). This approach also builds on previous work that suggests chirps may be more sensitive to measuring attentional modulation within the brainstem (Strauss et al., 2025). Compared with other methods, Cheech offers more acoustic control over the specific parameters used to elicit multiple, simultaneously recorded brain responses from naturalistic speech, all while participants perform a challenging listening task complete with spatial cues and attentional switching.

Here, we use Cheech-modified short stories presented both in quiet and in the presence of a competing talker to elucidate attentional effects within the brainstem through ABRs (i.e., wave V). Our results suggest wave V is most affected by the presence of another talker, but not selective attention to one talker over another. However, we demonstrate the relationship between ABR wave V and several higher-level speech-listening behaviors, including target word identification, comprehension, and subjective listening effort, suggesting the importance of subcortical processes to support successful SiN perception.

## Materials and Methods

### Participants

Twenty-nine participants were recruited for this study. To be eligible, prospective participants must be between 20 and 40 years old, speak English as their first language, and report no neurologic, psychiatric, or scalp conditions that directly affected their ability to understand speech or interfere with electrophysiologic measurements (e.g., epilepsy, certain strokes, ADHD, scalp problems, etc.). Participants were screened for visual acuity (i.e., better than 20/40 vision per Snellen chart), normal hearing (i.e., ≤25 dB HL air conduction thresholds between 250 and 8,000 Hz in both ears, no air-bone gaps >10 dB within 500–2,000 Hz, no asymmetries between the ears >20 dB at 500, 1,000, or 2,000 Hz), and cognitive abilities [>24 Montreal Cognitive Assessment (MoCA) score—consistent with an ongoing comparison study group of veterans; Nasreddine et al., 2005]. One participant withdrew from the study due to personal reasons; one participant was excluded due to technical difficulties during the EEG recordings, and two participants were excluded because they did not pass the hearing screener at the time of testing. This resulted in a final cohort of *N* = 25 total participants with the following demographics: average age = 22.88 ± 4.98 SD years old (range, 18–38 years old); 15 females and 10 males; *N* = 21 self-identified handedness right, *N* = 2 left, and *N* = 2 ambidextrous. Participants had an average of 16.86 ± 3.19 SD years of education and 4.20 ± 3.74 SD years of formal music training. Of those who reported some level of formal music training (*N* = 22), the average age they began music training was 10.05 ± 2.75 SD years old. The average MoCA score was 28.16 ± 1.46 SD (range, 25–30). Additionally, participants completed a version of the MacArthur Scale of Subjective Socioeconomic Status where they were asked to rate their perceived socioeconomic status (SES) relative to individuals across the US society using a ladder-rung rating from 1 (lowest SES) to 10 (highest SES; Adler et al., 2000). Mean subjective SES scores were 4.70 ± 1.30 SD and range 3–8. Average air conduction pure tone average thresholds were 6.80 ± 3.72 SD and 7.04 ± 4.47 SD for the right and left ears, respectively, indicative of normal hearing. All participants completed an informed consent as approved by the University of California, Davis Institutional Review Board and were compensated financially for their participation.

### Project overview

Each participant completed three sessions as part of a larger experiment investigating the influence of various factors on hearing across the lifespan: one 1 h audiologic exam, one 2 h behavioral session (i.e., a battery of cognitive and psychoacoustic tests), and one 2.5 h EEG session (i.e., our Cheech-based spatial attention speech-listening task followed by standard click ABRs). Several participants completed more than one session on the same day (i.e., either audiologic + behavioral or behavioral + EEG sessions), but a long break was offered in between sessions to reduce fatigue. Only a portion of the measurements and tests from the larger experiment are included in this analysis. The data presented here focus on brainstem activity from three out of six total blocks of the spatial attention task (i.e., during the EEG experimental session). The remaining three blocks are part of a separate study using different Cheech stimuli and thus were not included in the present analysis (Extended Data Fig. 1-1). Data from other components of the larger study will be analyzed in separate reports.

### Stimuli

The stimuli were three short fairytale stories available in the public domain (e.g., on www.librivox.com; plus one additional story for practice). For more information about story selection criteria in the current study (Extended Data Figure 1-1). The short stories were modified to add monosyllable color words as targets for the spatial attention experiment (e.g., red, green, blue, white, etc.) embedded within each story (e.g., “…with a narrow **blue** ribbon over her shoulders”). We recorded a male talker (native English speaker with a neutral American accent) reading the modified short stories aloud using a Shure KSM244 vocal microphone (cardioid polar pattern, high-pass filter of 18 dB per octave cutoff at 80 Hz) and Adobe Audition [sample rate, 48,000 Hz; 32 bit depth (float)] in a quiet, soundproof room. Silent gaps in the recordings >500 ms were shortened to 500 ms, similar to previous studies (Broderick et al., 2018; Teoh and Lalor, 2019; Teoh et al., 2022), and the trimmed recordings were then cropped to 7.5 min per story audio.

To produce a dual-talker condition, one story was pitch modulated using MATLAB STRAIGHT prior to the Cheech process (see below, Cheech; Extended Data Fig. 1-1; Kawahara et al., 2008; Kawahara and Morise, 2011). This manipulation allowed us to compare neural and behavioral responses for male versus female voices (between-story comparison) in addition to attended versus unattended conditions (within-story comparison; see below, Experimental conditions). Specifically, the female-intended story was modulated in pitch to an average F0 of 180 Hz and modulated in frequency characteristics to model a shorter vocal tract length, while the original (male voice) audio had an average F0 of ∼128 Hz. Modifying voice parameters in this manner meant speaker characteristics such as phrasing, intonation, and pacing were otherwise preserved because all stories were originally spoken by a single talker. The mono-talker story and the male dual-talker story were presented in the original, unmodified male voice only. Modifying one talker's F0 also provided the added benefit of a combined voice-sex + spatial release from masking which improved pilot test performance (Oh et al., 2021, 2022).

### Cheech

The short story auditory stimuli were then modified via a patented process called “Cheech” to evoke the auditory evoked potentials (Backer et al., 2019; Miller et al., 2020; Shehabi et al., 2025; Fig. 1). To produce “Cheech,” we used a level-specific CE-Chirp with a frequency band from 200 to 10,000 Hz designed for moderately loud intensity presentations (Chirp 1; Elberling et al., 2010) according to the cochlear–neural delay model by Elberling and Don (2008). The chirp audio file was upsampled from 30 to 48 kHz to match the speech recording sample rate.

**Figure 1.**
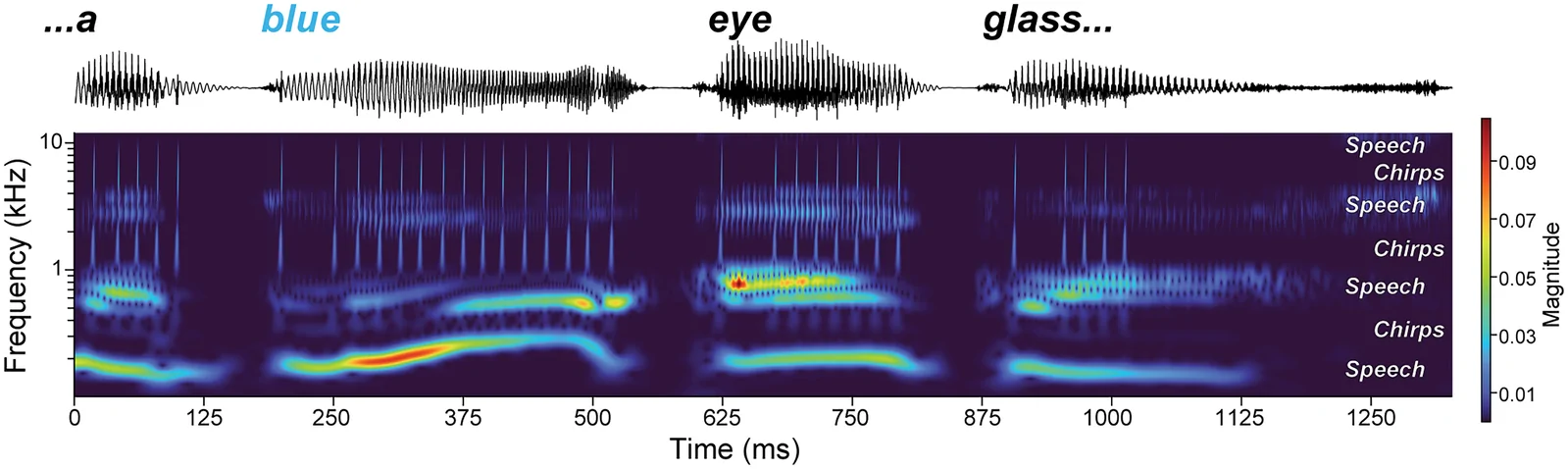
A brief segment of the Cheech stimuli. Top, Participants listened to the Cheech-modified short stories and pressed a button when they heard the target talker speak a color word (e.g., “blue”). Middle, Stimulus waveform and (bottom) spectrogram for the Cheech audio. Cheech interleaves speech and chirp frequency bands in a manner that preserves naturalistic speech qualities while evoking robust ABRs. See Materials and Methods for details. For more information on the short story stimuli and Cheech synthesis parameters, see Extended Data Figures 1-1 and 1-2, respectively.

10.1523/ENEURO.0272-25.2026.f1-1 Figure 1-1 Short story stimulus details. The stories used in this study were selected for content unlikely to be widely known, to avoid comprehension question recall based on modern renditions (i.e., popularized by children’s books or movies), and with story arc progress within a 7.5-minute block (i.e., rising action, climax, or falling action) to ensure sufficient and generally consistent content for probing comprehension. Thirteen stories were piloted on four raters who scored them according to factors such as interest and engagement levels, ease of understanding, grammar or writing style complexity, familiarity (i.e., low familiarity preferred), and suitability for an adult audience. Seven stories that scored highest across these factors were ultimately retained for the experiment. The seven stories were then divided into separate experimental conditions: two stories presented alone (“mono-talker”), two sets of paired stories (“dual-talker”), and one for initial practice. Given the constraint of naturalistic color word embeddings within each story, color words occasionally overlapped within the dual-talker pairings. The story pairs were determined according to those which had the fewest color word overlaps, and any overlapping color words were dropped from the behavioral task analyses to distinguish target story hits from masker story false alarms. As mentioned in the manuscript, three stories—one mono-talker story and a pair of dual-talker stories—were further modified during the Cheech process as part of a separate study. We do not include details or analyses of those stories here. The table below describes details for the remaining three experimental stories (i.e., one mono-talker story and a pair of dual-talker stories) plus the story used for practice. Story block presentation order was pseudo-randomized to ensure the same dual-talker story pairs were not heard back-to-back, and the order of all the blocks was counterbalanced across participants to reduce influence of any learning effects. Download Figure 1-1, DOCX file.

10.1523/ENEURO.0272-25.2026.f1-2 Figure 1-2 Cheech synthesis parameters. As mentioned in the Stimuli section, one voice within the dual-talker story pair was pitch shifted prior to the Cheech modification process to simulate a female speaker. Consistent with combined voice-sex and spatial release from masking outcomes observed in previous work (Oh et al., 2021, 2022), two distinct talker F0 s helped listeners better distinguish between the spatially-separated voices and more successfully complete the task during piloting. MATLAB STRAIGHT parameters associated with the female voice manipulation are listed below. Initially, female mono-talker stories were planned for the full experiment as well (i.e., resulting in nine stories instead of seven), but it became clear during piloting that additional stories would significantly increase the total testing time and lead to excessive fatigue, so these stories were ultimately dropped from the final experimental design. Download Figure 1-2, DOCX file.

In Cheech, some of the glottal pulse energy within predefined frequency bands are replaced with the synthetic chirp energy consistent with previously published protocols from our lab (Backer et al., 2019). Aligning the chirps with glottal pulse energy inherent in speech creates an acoustically fused auditory perceptual object with sufficient transient chirp activation to measure auditory brainstem through cortical responses while preserving naturalistic speech qualities and linguistic content (for analysis of cortical responses on this project, see Shehabi et al., 2025). First, periods of sufficient voiced power were identified; specifically, the audio was filtered from 20 to 1,000 Hz, and voiced periods at least 50 ms long were identified when the speech envelope between 20 and 40 Hz surpassed a threshold ∼28% of overall speech root-mean-square (RMS) amplitudes. The timing of the glottal pulses during voiced periods was determined by a speech resynthesis process using custom MATLAB code and the TANDEM-STRAIGHT toolbox that retains original speech frequency characteristics (Kawahara et al., 1999, 2008; Kawahara and Morise, 2011). The continuous speech was refiltered into alternating, octave-wide frequency bands of 0–250, 500–1,000, 2,000–4,000, 11,000–24,000 Hz with a 10th-order Butterworth filter, and chirp energy was constrained to frequency bands of 250–500, 1,000–2,000, 4,000–11,000 Hz by filtering the chirp with a fourth-order Butterworth filter. The chirp and speech bands in the alternating, interleaved octave-wide bands were then added together. In this manner, the glottal pulse retained in the speech bands and the energy within the chirp bands both contribute to cochlear activation, with more synchronized activity at predefined frequency regions where the chirps are present. Each mono track was then duplicated to form stereo audio. Audio files were normalized prior to the Cheech synthesis to ensure equivalent chirp amplitudes relative to the speech levels across each story, and the RMS amplitudes were rechecked across all Cheech-ed stories for equivalence after the Cheech process was completed.

Timing of the chirps varied with the natural fluctuations in the running speech, with special modifications to optimize measurement of the auditory ERPs. Specifically, when voicing energy was detected, chirps were inserted at a minimum of 18.2 ms apart (55 Hz), skipping glottal pulses that occur within the 18.2 ms window. The first chirp within a sequence is always followed by a minimum of 48 ms before the next chirp is presented, skipping glottal pulses in between, to produce a longer ISI that allows for improved evolution of middle- and late-latency responses (analyzed separately; Shehabi et al., 2025). For a summary of specific Cheech processing details, see Extended Data Figure 1-2.

### Spatial filtering and selective attention switching task

One goal of this study was to design a paradigm that mimicked real-life communication, including the ability to switch spatial attention to different talker locations. Spatial attention has been shown to modify neural activity associated with stream segregation (e.g., alpha power band lateralization), but these effects are often observed hundreds of milliseconds later than the ABRs explored here (Kerlin et al., 2010; van Diepen et al., 2016; Wöstmann et al., 2016; Teoh and Lalor, 2019). The results associated with spatial attention differences will be presented in a later publication. For completeness, we describe the details of the spatial attention switching task below, but for the sake of current analyses, results were collapsed across the attend-left and attend-right conditions.

The Cheech stimuli were spatially filtered via head-related transfer functions (HRTFs) to simulate audio at ±15° (SADIE II database; Armstrong et al., 2018). Spatial audio was mirrored by two symbols on two computer screens ∼15° to the left and right of the midline in front of the participant. A “+” symbol on one of the screens corresponded to a given target location .The “<” and “>” symbols were shown on the computer screen opposite from the target speech location (e.g., a “>” shown on the left screen and a “+” on the right screen indicated a the target voice located in the right auditory spatial field; Fig. 2). Consequently, “<” and “>” symbols indicated either the absence of another speaker for a given spatial field, as in the mono-talker condition, or the location of the masker story in the two-talker condition. The symbols were identical in size, color, and line lengths to maintain equivalent luminance for the eye tracking measurements (data not included here). The spatially filtered audio and visual symbols were then edited with custom MATLAB code to swap left and right spatial locations 75 times or approximately every 6 s (7.5 min ÷ 75 switches = 6 s; switches assigned per random distribution of 6 ± 1 s). The visual icons switch screens instantaneously at each switch time while the crossover fade time for the audio was 35 ms, beginning at the onset of a switch cue (Fig. 2). During piloting, this crossover time was an appropriate balance between minimizing “pops” and other artifacts from cutting the audio across left and right audio tracks while also minimizing the perception of a gradually moving sound between spatial locations (i.e., a sudden jump from one side to the other without introducing robust distortions). In this manner, our spatial attention switching task mimicked listening scenarios with multiple alternating targets, as if perceptually following a conversation between two spatially separated talkers.

**Figure 2.**
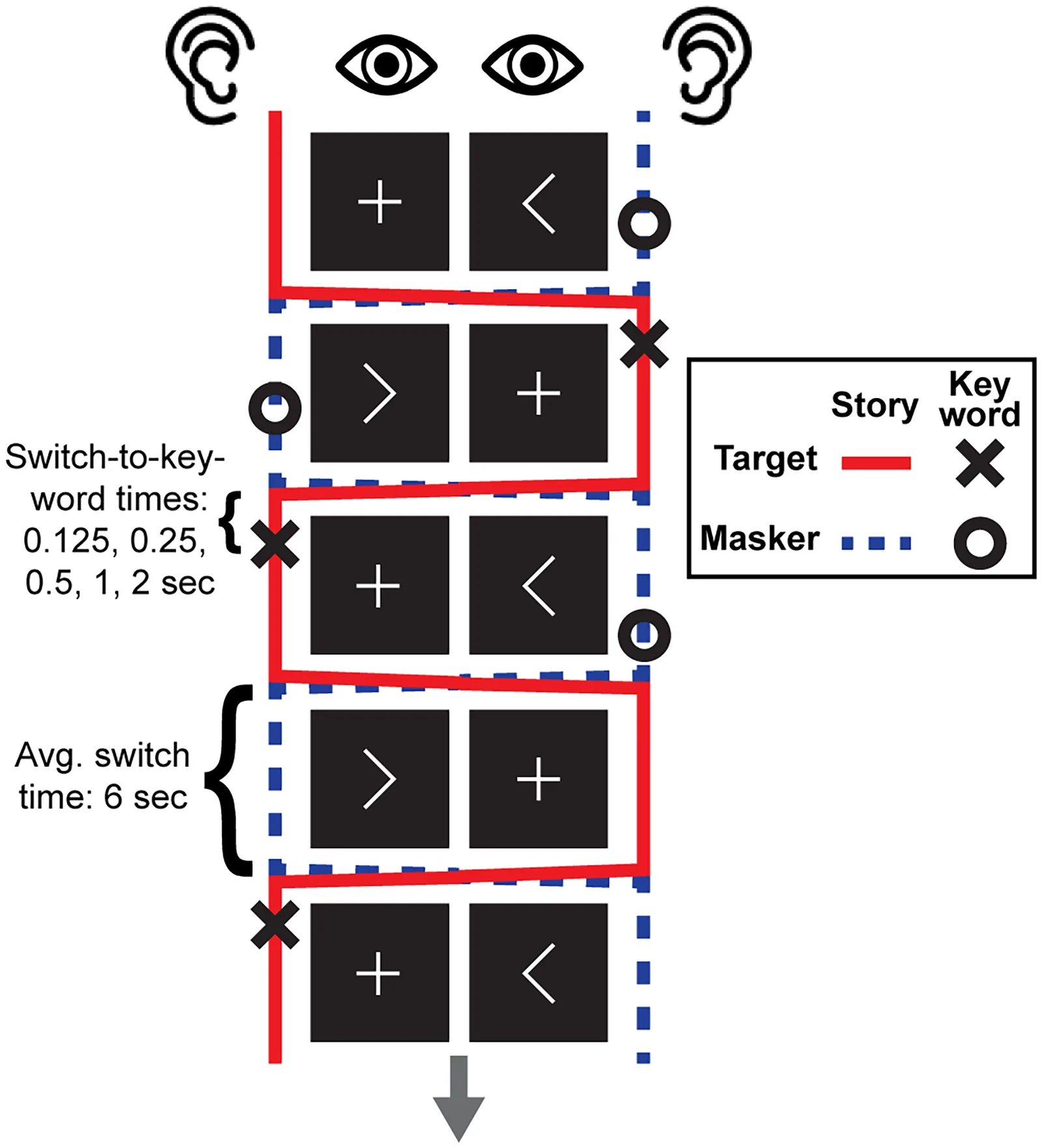
Experimental paradigm. Participants are instructed to attend to the target speech (red line) and press a button when a color word is spoken (“X”). They must ignore color words spoken by the masker talker when present (i.e., dual-talker condition; “O”). The target visual cue and voice alternate between approximately ±15° in the visual and auditory spatial fields on average around every 6 s (see Materials and Methods). Figure modified from Shehabi et al. (2025).

Color words were randomly assigned to one of five possible onset categories between 0.125 and 2 s from the switch-to-color-word-onset (i.e., 0.125, 0.25, 0.5, 1, and 2 s, five color words per condition; Fig. 2). These categories were established after preliminary piloting to assess whether “attentional blinks” from switching left to right spatial attention following the cue (and vice versa) impaired color word detection across participants; these data will be presented in a subsequent paper. Onset timings of the color words were determined by Gentle, an audio forced aligner (https://github.com/lowerquality/gentle). The closest preceding switch time was replaced by the assigned switch-to-color-word-onset, resulting in 50 random switches and 25 switch-to-color-words (i.e., 33% probability of a target color word onset within 2 s of a switch). The 25 target words were balanced across left-to-right and right-to-left switches within a story (minus one remainder). A few target story switch times were also manually adjusted to ensure that no masker color word occurred during a switch in the dual-talker condition (i.e., masker color word onset was not between −0.5 and 0.1 ms from a target story-driven switch time). Any overlapping color words across the target and masker stories were dropped from the behavioral task analyses, so behavioral responses to target (hits) and masker color words (false alarms) would not be conflated (*n* = 4; see below, Behavioral outcome measures and analysis).

### Experimental conditions

The short stories in this analysis were used in separate experimental conditions: one story presented alone (“mono-talker”), a pair of stories (“dual-talker”), and one additional story for initial practice which was not included in subsequent analyses. Within the dual-talker condition, each story was heard twice—once as the “target story” and once as the “masker story” within the pair. This experimental manipulation thus allowed us to measure effects of directed spatial attention because the same audio was presented and participants were instructed to either “attend” or “ignore” the story, respectively. The mono-talker story was only presented once. The target story always started on the left side, whether presented alone (mono-talker) or in the presence of a masker (dual-talker). The order of stories and conditions was counterbalanced with a Latin square design across participants to reduce potential sequence-dependent learning effects. In the full experiment with an additional three stories, no story pair was heard back-to-back to reduce short-term learning effects as well (Extended Data Fig. 1-1).

### Stimulus presentation equipment

Participants were seated in a soundproof, electromagnetically shielded booth (ETS Lindgren), ∼176 cm from two computer monitors. The Dell UltraSharp monitors had a refresh rate of 60 Hz, and screen settings were left on standard factory defaults. Stimulus presentation, including both audio and visual, was controlled by custom MATLAB code using Psychtoolbox (Brainard, 1997; Pelli, 1997). Auditory stimuli were routed from a Hammerfall audio card to an audio interface (RME Fireface UFX II) and then to a headphone driver (RME ADI-2DAC FS) which drove ER-2 insert earphones (Etymotic Research/Interacoustics). The ER-2 inserts were electromagnetically shielded to reduce EEG artifacts (Campbell et al., 2012). Cheech stimuli were presented at 75 dB SPL because conversational speech levels are often moderately elevated in the context of noisy settings (Rusnock and Bush, 2012). Clicks for the separate ABR recordings were presented at 80 dB SPL. Stimuli were calibrated using a Larson-Davis PRM902 preamp and 2221 power supply, B&K 4157 coupler, and C-weighted average.

### Procedure

The order of events within each block is as follows: 1 min of brief rest (for resting-state EEG analysis), 7.5 min of the spatial attention listening task, five comprehension questions, six subjective task load questions (Hart and Staveland, 1988), a flash reaction time task (five short trials), and then a break. During the 1 min rest period, participants were instructed to remain still with their eyes open. During the spatial attention listening task, participants listened to the short stories, following the target story and corresponding visual cue as they switched between left and right relative spatial locations (Fig. 2). They were instructed to press the spacebar when they heard the embedded color words within the story. In dual-talker condition blocks, participants were told to ignore color words from the masker story and only press the spacebar for target story color words. The audio was immediately followed by a series of five multiple-choice comprehension questions with four possible answer choice options (chance, 25% accuracy). Comprehension questions were balanced for difficulty (not too hard nor too easy; piloted for accuracy on 3–5 individuals per story), story time relevant for the question (i.e., early vs late in the 7.5 min story segment), and memorization versus inductive reasoning probed by the answers. Next, participants completed the NASA Task Load Index, a measure of subjective task-related workload or effort, modified for computerized data collection using a sliding scale selection tool (Hart and Staveland, 1988). To measure general mental fatigue across the blocks, we used a psychomotor vigilance-based visual reaction time task (Wilkinson and Houghton, 1982; Dinges and Powell, 1985; Basner and Dinges, 2011; Hornsby, 2013). Participants were instructed to watch the crosshairs shown at the center of the computer screen and press the spacebar as quickly as possible when a white circle appears on screen. The circle flashed on screen for 40 ms across five trials. Finally, participants were offered a break before starting the next block. The entire experiment presentation was controlled by custom MATLAB and Psychtoolbox code.

### Behavioral outcome measures and analysis

Selective attention listening was assessed by correct identification of color words embedded in the target story (hits). A “hit” was defined as pressing a designated keyboard button within 2 s after the onset of a target word. Any pairs of color words within ±2 s onset across the target and masker stories in the dual-talker condition were excluded from analyses, resulting in four overlapped color words dropped from each story in the behavioral task analyses for the pair of stories used here. Specifically, the relevant measures were the proportion and corresponding reaction times of correct hits out of the total possible, nonoverlapping target words (*n* = 25 in the mono-talker condition; *n* = 21 per story in the dual-talker condition). Behavioral outcomes measured after each story concluded were comprehension question accuracy (i.e., total percentage correct out of five questions), task load effort scores (i.e., average NASA task load index scores across six questions, where higher scores means greater perceived task load; Hart and Staveland, 1988), and average reaction times on the vigilance-based flash task in a given block.

### EEG recordings

EEG recordings were obtained using a BioSemi ActiveTwo system (BioSemi B.V., www.biosemi.com) in an electrically shielded booth with a sample rate of 8,192 Hz recording data from 72 electrodes: 64 channels following the International 10–10 standard with four additional channel pairs around the ears (earlobe, mastoid, lower posterior, posterior to the earlobe and inferior to the mastoid; upper anterior, superior–anterior to the tragus near the zygomatic arch). Electrode offsets from all channels were measured to be below 20 µV relative to the Common Mode Sense electrode using the ActiveView2 software. Data were recorded using the open-source Lab Streaming Layer (LSL) software (https://github.com/sccn/labstreaminglayer) as XDF files on a desktop computer running Windows 10.

A pair of channel outputs containing trigger events was routed from the audio interface to a pair of Brain Products StimTraks for monitoring triggers associated with the target and masker stories, respectively, which then convert the audio trigger signals to trigger pulses sent to the TriggerBox (Brain Products). This setup permitted audio playback synchronized with chirp triggers embedded during the Cheech process with minimal latency delays (see Cheech section); such near-simultaneous synchronicity is crucial for measuring rapid, lower-level ABRs during the listening task. Triggers controlled by Psychtoolbox (i.e., participant responses, block sequence triggers, spatial location switches, etc.) were also routed to the TriggerBox via the computer parallel port. The TriggerBox then passed both triggers from the parallel port and the StimTraks to the Biosemi signal receiver box.

### EEG preprocessing

EEG were preprocessed using MATLAB 2021b (MathWorks) using a combination of EEGLAB functions (Delorme and Makeig, 2004), ERPLAB functions (Lopez-Calderon and Luck, 2014), and custom MATLAB code. Data were first imported into EEGLAB with events extracted from the trigger channel in the XDF file using custom MATLAB code. Given the large number of tightly packed event codes for our experimental design (>130,000/participant at an average rate of one event every 20 ms), event codes from the three separate streams (parallel port and two StimTraks, described above) occasionally arrived at the TriggerBox simultaneously, resulting in correctable event code errors. Custom code was used to check the recorded events against the expected events for a given condition to add missing events, remove extra events, and adjust event latencies for any overlapped event. A second set of corrections were then applied to adjust for latency differences between the acoustic signal and the event times. These corrections took into account a 1 ms delay due to audio travel time from the insert earphone tubes, a 1.973 ms delay to the audio signal due to the HRTF process, a 0.541 ms delay to the chirp triggers to account for a difference between the expected chirp timing from the Cheech process and the chirp onsets, and a 1 ms delay in one of the StimTrak channels caused by the TriggerBox hardware.

Data were then pruned to retain only rest periods and attentive listening task periods along with a 10 s buffer on both sides of each selected period. Following pruning, each section of the data was DC corrected, and a second-order 0.1 Hz high-pass Butterworth filter was applied to remove slow drifts. We then referenced the data to electrode Cz for independent component analysis (ICA). Even though our data were collected in an electromagnetically shielded booth, we occasionally found 60 Hz line noise in some channels. To minimize the effects of cleaning line noise, only channels with detected line noise were cleaned (mean = 4.4 channels ± 7.56 SD, range for a given participant = 0–39 channels). This was carried out by first checking for line noise using a frequency tagging approach. Spectra were calculated across all frequencies for each channel. For each frequency, the average spectra of four neighboring frequencies except those immediately adjacent (*x* − 2, *x* − 3, *x* + 2, *x* + 3) were subtracted from the spectra for each frequency. If the neighbor corrected power at 60 Hz or any of its first four harmonics (120, 180, 240, 300Hz) was greater than eight standard deviations calculated from the corrected power from remaining frequencies, that channel was cleaned of line noise using the Cleanline plugin (https://www.nitrc.org/projects/cleanline). This process was repeated until no further line noise was detected up to a maximum of five repetitions.

To clean the data of prominent artifacts such as eyeblinks, eye movements, and heartbeat signals, we used ICA following the methodology outlined by Luck (2022) which improves ICA performance and greatly reduces the required processing time, compared with more traditional approaches. This process involved making a copy of the processed data (the ICA dataset), downsampling and precleaning it of prominent (noneye and heart) artifacts, applying ICA to select and remove eye and heart artifacts, and then transferring the calculated component weights back to the original dataset. Specifically, we first downsampled the data to 256 Hz to speed ICA processing; then we inspected and rejected bad channels by eye (mean = 3.72 ± 2.13 SD channels; range, 0–9 channels). Following channel rejection, we applied a noncausal eighth-order Butterworth bandpass filter with cutoffs at 1 and 50 Hz. Further cleaning was then undertaken using ERPLABs continuous artifact rejection function to remove sections of data with significant movement and muscle amplitude artifacts, the presence of which can hinder ICA’s ability to isolate eye and heart components (mean amount of data in seconds removed for ICA = 129.19 ± 86 s SD or 3.6 ± 2.35%; range, 4.98–298.79 s or 0.14–7.96%). The rejection criterion operated on a 1 s sliding window with a 0.25 s step size and an initial threshold of 200 µV. As we wanted to retain eye activity for ICA, frontal channels that contain the largest eye-related activations (FP1, FPz, FP2, AF7, AF3, AFz, AF4, AF8) were excluded from the rejection threshold. We then inspected and adjusted the threshold level for each dataset (mean = 220 ± 28.87 SD µV; range, 200–300 µV) until only those sections of data with large muscle or movement artifacts were rejected while any eyeblink or eye movement-related artifacts remained. To further speed the ICA processing time, the number of input channels was reduced from an average of 67 channels to the first 32 principal components using principal component analysis, then ICA was performed using the infomax algorithm. Eyeblink, eye movement, and (when present) heart artifact components were manually marked for rejection (mean = 2.8 ± 0.65 SD components; range, 2–4 components). Following ICA processing, we prepared the original dataset for transferring component weights by first removing the same bad channels marked for removal in the ICA dataset. We then transferred the calculated ICA weights from the ICA dataset to the original dataset and rejected the marked artifact components in the original dataset. Thus, any eye and heart artifact components were removed from the dataset with the original sample rate (and without the aggressive precleaning done to prepare the ICA dataset).

Once the data were cleaned with ICA, we applied a noncausal eighth-order Butterworth bandpass filter with cutoffs at 100 and 1,500 Hz and then re-referenced to the average of the left and right earlobes. If one of the earlobe channels had been rejected as a bad channel, the average of the left and right mastoid was used instead. Activity at channel Cz (the previous reference) was then recalculated and added back to the dataset. We then interpolated any rejected bad channels using spherical interpolation.

Epochs were extracted between –2 and +15 ms of chirp onset for all conditions with baseline correction from –2 to 0 ms. The number of epochs was balanced so that each condition had the same number of chirps both in total and from each spatial location (left, right), resulting in each condition (mono-talker male, dual-target male, dual-target female, dual-masker male, dual-masker female) having 7,886 epochs per condition equally distributed across left and right presentation (3,943 epochs from both left- and right-side presentations per condition). The smallest number of chirps on any one side for all conditions was calculated, and epochs were randomly sampled so all conditions were balanced. Random sampling and balancing the number of trials reduced possible transient effects of attention-driven modulations during or immediately after a spatial attention switch. Additionally, balancing of the number of epochs for each condition was done to avoid any systematic condition differences due to a difference in SNR caused by some conditions having more chirps than others. This difference was primarily due to the female-voiced stories, where more frequent glottal pulses (driven by a higher F0) resulted in ∼9% more chirps in the female-voiced stories compared with the male-voiced stories. Additional differences in total chirp events were due to an ∼3% variation of chirps across stories (i.e., subtle differences in voicing and pausing). Given evidence for left–right asymmetries in speech processing and neural encoding (e.g., “right-ear advantage”; Ahonniska et al., 1993; Jerger and Martin, 2004; Hornickel et al., 2008; Keefe et al., 2008; Bidelman and Bhagat, 2015), we also balanced the number of chirps across stimulus presentation side for each condition to ensure results were not driven by unequal ear-based SNR or ear presentation differences in the EEG measures (∼5% variation in the of number of chirps across sides within each story). Epochs with artifacts (e.g., muscle activity) were then removed with a two-step process: first, any epochs with activity outside the threshold of ±100 µV were rejected, and then any remaining epochs whose activity was outside of six SD for a single channel or two SD across all channels were eliminated (mean = 9.85 ± 6.24% SD of rejected epochs; range, 3.65−29.96% rejected epochs across individuals and conditions).

ABR wave V peak amplitudes and latencies were extracted at channel Cz from the average amplitude over time across the remaining epochs for each condition for each participant. Wave V latency measures were extracted by fitting a local positive peak in a window between 5 and 8 ms post chirp-onset. Wave V amplitude was computed as the peak-to-peak amplitude between the local positive peak within 5–8 ms and the subsequent local negative peak between 7 and 11 ms. The measurement windows were determined based on visual inspection of all individual wave Vs for each condition. In cases in which more than one local peak was seen in the wave V waveform inside the measurement window, the values from the peak with the greatest amplitude were taken.

### Statistical analyses

Unless otherwise specified, mixed-effects linear regression models and repeated-measures ANOVAs were used to analyze the data in SAS Studio (v.3.82). All regression models included a random intercept of subject to account for inherent variability present between individuals and improve generalizability of the results. Outcome measures were ABR wave V amplitudes and latencies for the neural models, and the behavioral metrics (i.e., target word identification accuracy, target word identification reaction time, comprehension question accuracy, subjective task effort ratings) were included as dependent variables for the behavior-only analyses as well as the brain–behavior comparisons. Independent variables were characterized by the condition of interest (i.e., one vs two talkers; target vs masker; also main effects and interaction terms with male vs female voices where appropriate). When interactions were not significant in the full model, they were removed using a backward selection procedure, and the remaining simplified model variables are reported in the results as described below. All post hoc comparisons were corrected using Tukey adjustment. Influential outliers were identified by externally studentized marginal residuals (also known as jackknife studentized residuals) exceeding ±3 and Cook's *D* values >4/*n*, where *n* equals the number of observations in the dataset, as calculated by the PROC MIXED statement in SAS (Anon, 2026). Influential outlier observations were excluded from the reported results (i.e., one observation each for amplitude condition effects, amplitude masking effects, latency attention effects, and behavioral identification accuracy; see results below). Any influential outliers found for either the neural or behavioral data separately were also excluded from the relevant behavior–behavior and brain–behavior analyses. Non-normality of the dependent measures was evaluated using the Shapiro–Wilk test. A Box–Cox transformation procedure was performed on non-normal variables; specifically, the first convenient power parameter within the lambda confidence interval was used to select a transformation method (cf. optimal power parameter; specified by the “convenient” *t*-option). However, data transformations did not alter results for any non-normally distributed data, so the nontransformed results are reported below. Effect sizes are also included for the *F* statistics and post hoc *t* statistics using partial eta-squared (*η^2^p*) and Hedge's *g* (average), respectively (Uanhoro, n.d.). In the case of an excluded influential outlier, Hedge's *g* was computed using the mean values of the remaining balanced pairs, whereas pairwise, Tukey-corrected post hoc comparisons relied on least squares means.

## Results

### ABR condition effects

To evaluate general listening condition effects on the ABR wave V during the selective attention task, we compared Cheech responses for all conditions individually (i.e., mono-talker male target, dual-talker male target, dual-talker male masker, dual-talker female target, and dual-talker female masker) using mixed-effects regression analysis. Overall, the model suggested at least one listening condition differed across wave V amplitudes (*F*_(4,95)_ = 6.31; *p* = 0.0002; *η^2^p* = 0.2099) and latencies (*F*_(4,96)_ = 3.86; *p* = 0.0059; *η^2^p* = 0.1385). Tukey-adjusted post hoc comparisons revealed larger amplitudes for the mono-talker (male) condition compared with the dual-talker male target (*t*_(95)_ = 3.84; *p* = 0.0020; *g* = 0.6230), male masker (*t*_(95)_ = 4.58; *p* = 0.0001; *g* = 0.7280), and female target (*t*_(95)_ = 3.65; *p* = 0.0039; *g* = 0.4601) conditions individually (cf. borderline female masker: *t*_(95)_ = 2.6810; *p* = 0.0644; *g* = 0.4274). Additionally, faster latencies were observed for the mono-talker condition compared with the dual-talker female target (Tukey-adjusted *t*_(96)_ = −3.00; *p* = 0.0276; *g* = −0.4751) and dual-talker male masker (Tukey-adjusted *t*_(96)_ = −3.61; *p* = 0.0043; *g* = −0.5815).

### Masking and SiN effects: single- versus dual-male talker

Though the condition effects are highly suggestive of a masking effect on the ABR, characteristic differences between the male and female voices may potentially confound the results. As mentioned in the methods, the study design included only a male speaker for the mono-talker condition (cf. female mono-talker) due to time constraints and excessive listening fatigue. A secondary mixed-effects regression analysis was therefore conducted to isolate the most similar, salient masking contrast: male mono-talker versus male dual-talker (target only). Consistent with the full model results, the male dual talker was associated with reduced wave V amplitudes (*F*_(1,23)_ = 26.39; *p* < 0.0001; *η^2^p* = 0.5343) and delayed wave V latencies (*F*_(1,24)_ = 7.60; *p* = 0.0110; *η^2^p* = 0.2405) compared with the male mono-talker condition. Collectively, these results suggest poorer brainstem encoding for stimuli in the presence of a competing masker (i.e., smaller amplitudes and delayed latencies; Fig. 3).

**Figure 3.**
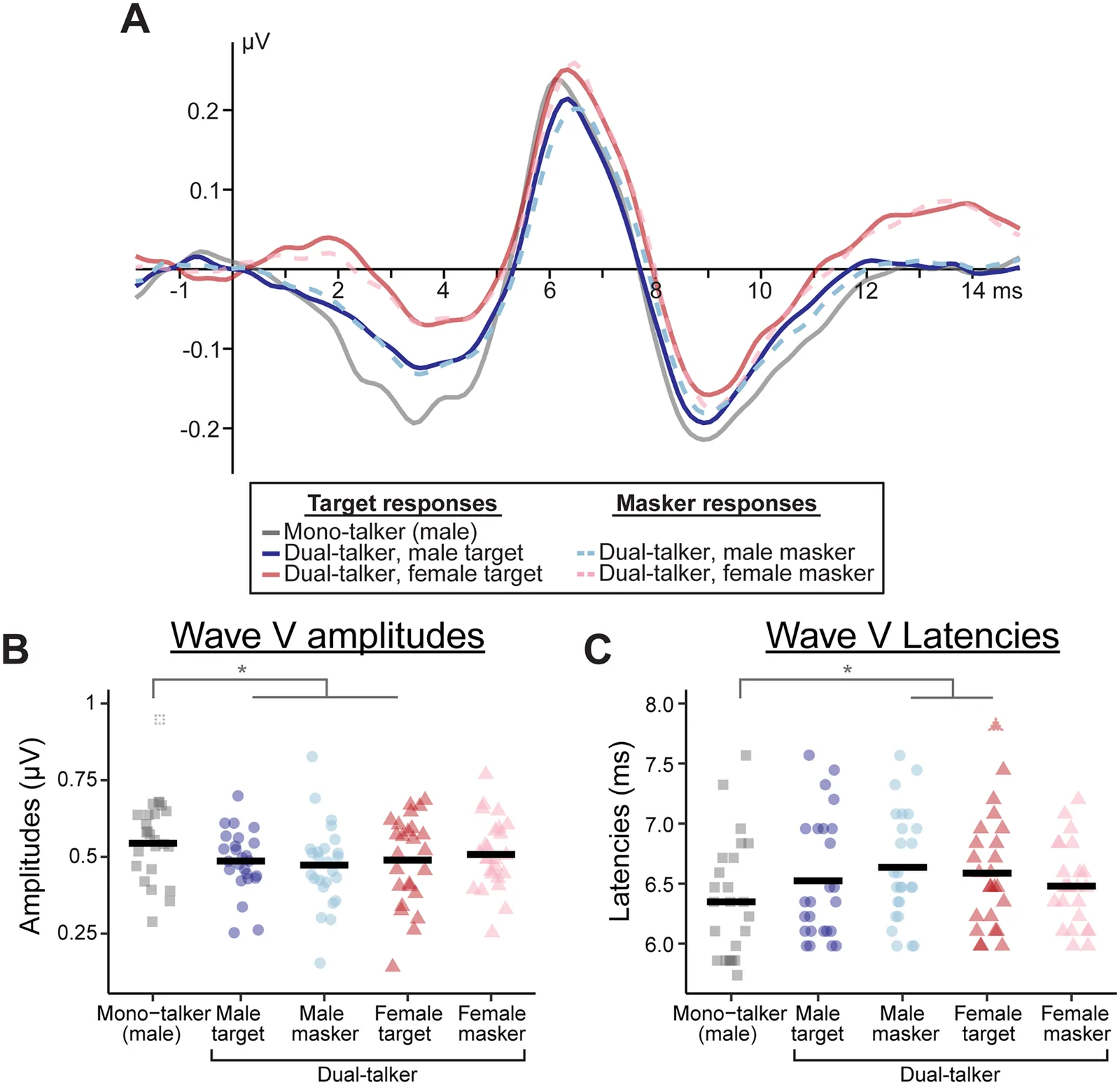
***A***, ABR waveforms evoked by the Cheech chirps across target and masker conditions show wave V differences with the presence of another talker (SiN effects) as well as directed selective attention (target vs masker). ***B***, Wave V amplitudes were significantly larger for the mono-talker condition compared with the dual-talker male target, male masker, and female target conditions (compare Tukey-adjusted *p* = 0.0644 for female masker). ***C***, Wave V latencies were faster for the mono-talker condition compared with the dual-talker male masker and female target conditions. Figure icons depict individual subject observations while the bold black line indicates the mean. The white-filled icon with a dashed gray outline indicates an influential outlier that was excluded from wave V amplitude mean calculations and pairwise comparisons regarding overall condition effects and masking effects, whereas the red-filled icon with a dashed outline indicates an influential outlier for the wave V latency attention effects analysis only (thus included in mean and pairwise condition effects shown here; see text). *p < 0.05.

### Attention effects: target versus masker

We next wanted to evaluate possible effects of selective attention on the ABR wave V by comparing target and masker conditions. Although the mono-talker male could be considered a target condition itself, even in the absence of a masker talker, it is possible that including this condition in the models introduces a confound in the statistical models due to the larger amplitudes and faster latencies compared with the dual-talker conditions. Like the masking effects results above, we thus performed a secondary linear mixed-effects regression analysis on a subset of the data that only included the male and female dual-talker conditions (i.e., main effects of target vs masker, talker sex, and their interaction). Neither target versus masker nor male versus female voice main effects were significant (*F*_(1,72)_ = 0.03 and 2.02; *p* = 0.8585 and 0.1593; *η^2^p* = 0.0004 and 0.0273, respectively) nor was their interaction (*F*_(1,72)_ = 1.36; *p* = 0.2480; *η^2^p* = 0.0185). Similar results were observed for wave V latencies after removal of one influential outlier observation (interaction term, *F*_(1,71)_ = 2.61; *p* = 0.1103; *η^2^p* = 0.0355; target vs masker main effect, *F*_(1,71)_ = 0.33; *p* = 0.5690; *η^2^p* = 0.0046; male vs female voice main effect, *F*_(1,71)_ = 2.04; *p* = 0.1574; *η^2^p* = 0.0279). Thus, we cannot conclude from these results any attention effects on subcortical processing.

### Behavioral performance

One-way repeated-measures ANOVAs were used to evaluate differences in behavioral performance metrics across conditions (i.e., mono-talker male, dual-talker male, and dual-talker female). Selective attention performance was measured behaviorally as identification accuracy and corresponding reaction times for color words embedded in the target story (Fig. 4). Color word identification accuracy significantly differed across all three experimental conditions (*F*_(2,47)_ = 17.55; *p* < 0.0001; *η^2^p* = 0.4275; Fig. 4*A*). Specifically, both the mono-talker male and dual-talker male conditions were associated with higher identification accuracy than the dual-talker female (*t*_(47)_ = 4.8848 and *t*_(47)_ = 5.3903; *g* = 0.8387 and *g* = 0.8612, respectively; both Tukey-adjusted *p* < 0.0001). The two male talker conditions were not significantly different (*t*_(47)_ = −0.5133; *p* = 0.8653; *g* = −0.0901), suggesting a slight behavioral identification accuracy decrement specific to the female-voiced story, not necessarily with the general presence of a competing talker. Meanwhile, target color word reaction times also differed across conditions (*F*_(2,48)_ = 10.02; *p* = 0.0002; *η^2^p* = 0.2945). Specifically, faster reaction times were observed for the mono-talker block compared with the dual-talker female (*t*_(48)_ = −4.4761; *p* = 0.0001; *g* = −0.6789; Fig. 4*B*). Although the other Tukey-adjusted post hoc comparisons were not significant, reactions times trended toward faster reaction times for the mono-talker male compared with the dual-talker male (*t*_(48)_ = −2.2442; *p* = 0.0739; *g* = −0.3433) and for the dual-talker male compared with the dual-talker female condition (*t*_(48)_ = −2.2318; *p* = 0.0760; *g* = −0.3178).

**Figure 4.**
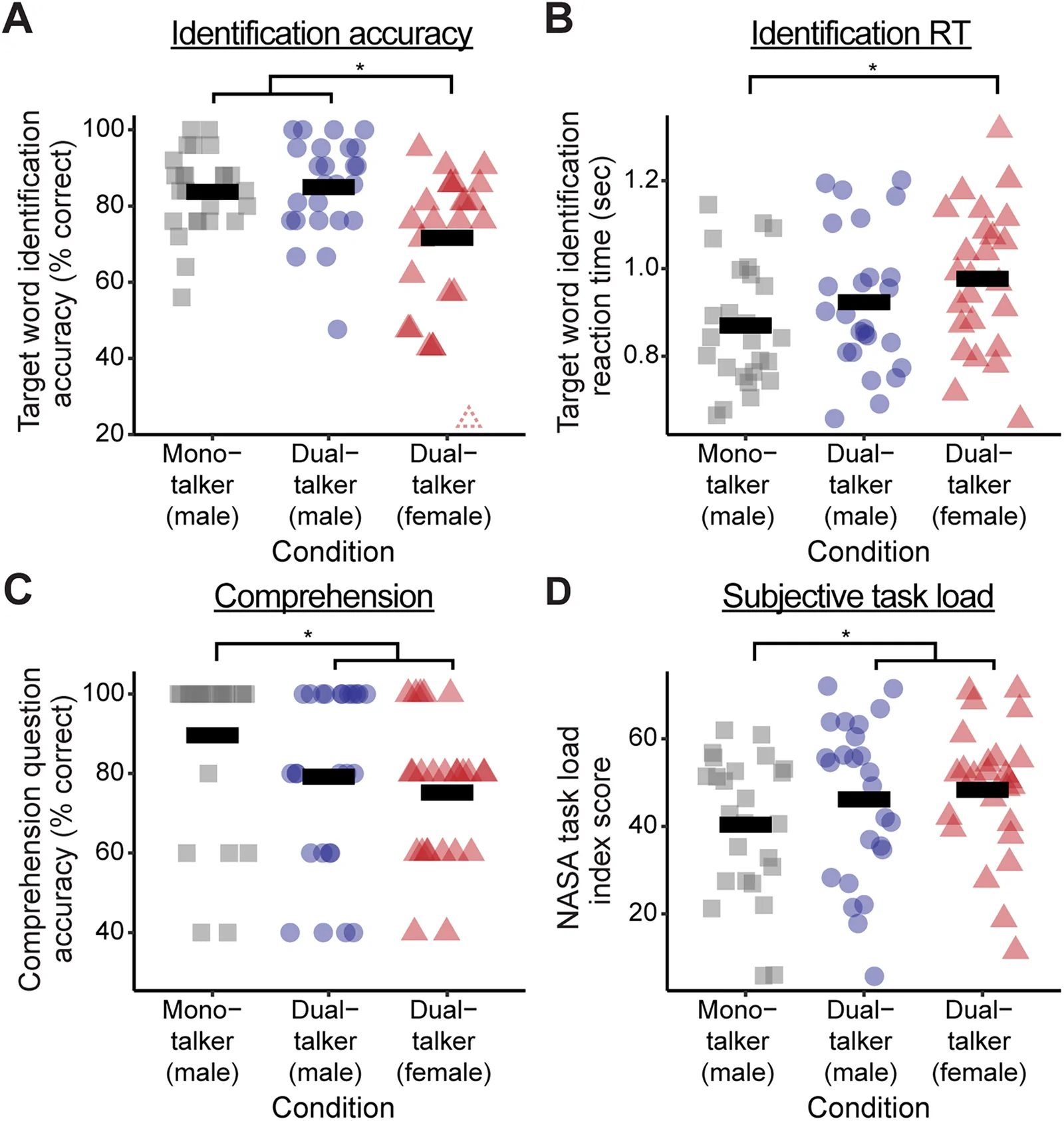
Behavioral results for color word identification accuracy (***A***), identification reaction times (***B***), narrative comprehension (***C***), and ratings of subjective task load or effort (***D***). Figure icons depict individual subject observations, while the bold black line indicates the mean. The white-filled icon with a dashed red outline indicates an influential outlier that was excluded from identification accuracy mean calculations and pairwise comparisons. *p < 0.05. See Extended Data Figure 4-1 for a summary of inter-relationships between the four behavioral metrics.

10.1523/ENEURO.0272-25.2026.f4-1 figure 4-1 Associations between the four behavioral metrics. Each figure plots the individual observations across the three target conditions (mono-talker male, dual-talker male, and dual-talker female) and the regression line depicts the relationship between the behavioral variables. Specifically, better behavioral performance was characterized by higher identification accuracy, shorter identification reaction times (RT), higher comprehension accuracy, and lower subjective task load or effort (NASA task load index scores). Mixed effects regression results for the main fixed effect are shown at the top of each figure plot. The influential outlier identified in Figure 4A was excluded from the analyses and thus not shown on the current plots. Shaded region = ± 95% confidence interval. Download figure 4-1, TIF file.

Following the listening task, participants were asked to complete several comprehension questions to probe their understanding of the short story segments. Response accuracy for comprehension questions was higher for the mono-talker condition compared with either dual-talker condition (overall *F*_(2,48)_ = 5.97; *p* = 0.0048; *η^2^p* = 0.1991; Tukey-adjusted mono-talker vs dual-talker male *t*_(48)_ = 2.4175; *p* = 0.0501; *g* = 0.4696 and mono-talker vs dual-talker female *t*_(48)_ = 3.3473; *p* = 0.0045; *g* = 0.7384; Fig. 4*C*). No difference was observed between the male- and female-voiced dual-talker conditions (*t*_(48)_ = 0.9298; *p* = 0.6243; *g* = 0.1907). Participants then completed the NASA Task Load Index questionnaire (Hart and Staveland, 1988) as a measure of their subjective task load or effort. The mono-talker story was associated with lower scores (i.e., less perceived task load) compared with either dual-talker condition (overall *F*_(2,48)_ = 8.11; *p* = 0.0009; *η^2^p* = 0.2526; dual-talker male, *t*_(48)_ = −2.8322; *p* = 0.0182; *g* = −0.3257; dual-talker female, *t*_(48)_ = −3.8958; *p* = 0.0009; *g* = −0.4971), but the dual-talker blocks did not significantly differ from each other (*t*_(48)_ = −1.0636; *p* = 0.5410; *g* = −0.1262; Fig. 4*D*). Collectively, the results suggest that the dual-talker scenario was associated with both greater perceived effort (task load) and impaired comprehension abilities.

Relationships between behavioral measures were also evaluated using linear mixed-effects regression models. All four behavioral outcomes were associated with each other (all *p*’s < 0.05). For further details and individual statistical analyses, see Extended Data Figure 4−1. As might be expected, better behavioral performance was characterized by higher identification accuracy, faster identification reaction times, higher comprehension question accuracy, and lower effort ratings.

### Brainstem–behavior relationships

We first analyzed mixed-effects regression models with main effects and interactions of directed attention (i.e., target vs masker), talker sex, and wave V measures to predict behavioral outcomes (i.e., separate models for latencies and amplitudes). No three- or two-way interactions were observed between either wave V measure and directed attention or talker sex variables (all *p*’s > 0.05). The only two-way interactions between talker sex and directed attention observed in the full model simply replicated findings previously reported in the Behavioral performance section.

The models were then reanalyzed to assess only main effects of ABR wave V amplitudes and latencies separately on behavioral outcomes (Fig. 5 and Table 1). These exploratory analyses broadly investigate whether wave V across all the conditions relates to behavioral outcomes rather than being tied to specific condition effects. In these simplified analyses, faster wave V latencies were associated with better target word identification accuracy (*F*_(1,97)_ = 5.35; *p* = 0.0228; *η^2^p* = 0.0523), better comprehension question accuracy (*F*_(1,99)_ = 4.73; *p* = 0.0320; *η^2^p* = 0.0456), and lower subjective task load (*F*_(1,99)_ = 19.28; *p* < 0.0001; *η^2^p* = 0.1630). Latencies alone did not correlate with target word identification reaction times (*F*_(1,99)_ = 1.06; *p* = 0.3050; *η^2^p* = 0.0106). Furthermore, larger wave V amplitudes were associated with faster target word identification reaction times (*F*_(1,98)_ = 7.09; *p* = 0.0091; *η^2^p* = 0.0675) but not target word identification accuracy (*F*_(1,96)_ = 1.33; *p* = 0.2524; *η^2^p* = 0.0137), comprehension question accuracy (*F*_(1,98)_ = 0.41; *p* = 0.5223; *η^2^p* = 0.0042), nor subjective task load (*F*_(1,98)_ = 0.34; *p* = 0.5587; *η^2^p* = 0.0035, respectively). Collectively, these results indicate a strong relationship between wave V and measures of successful speech (-in-noise) perceptual abilities. Specifically, wave V encoding strength (i.e., amplitudes) related to behavioral response time, whereas neural encoding speed (i.e., wave V latencies) related to target word identification, narrative comprehension, and perceived effort.

**Figure 5.**
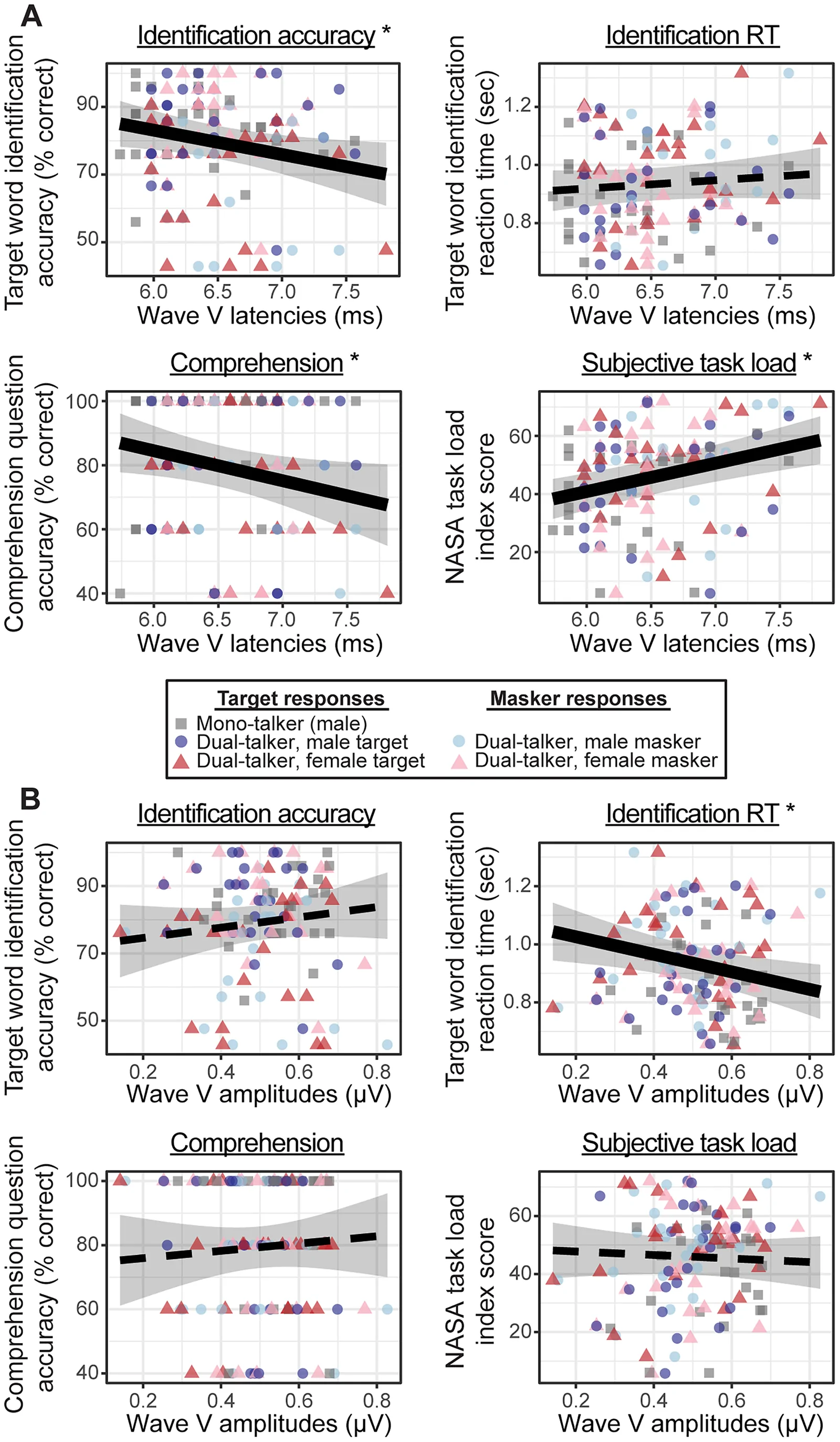
Associations between ABR wave V (***A***) amplitudes and (***B***) latencies and each of the four behavioral metrics. The figures show individual observations across all subjects and conditions with the regression line depicting the brain–behavior relationship. Bolded regression lines and an asterisk in the plot title indicate significant fixed effect results (*p* < 0.05), whereas dotted lines are not significant (*p* > 0.05). See Table 1 for regression results. The influential outliers identified in Figures 3*B* and 4*A* were excluded from these analyses and thus not shown on the current plots. Shaded region represent ±95% CI.

**Table 1.** Brainstem–behavior regression model results for wave V main effects

Wave V predictor	Outcome	*β* estimate	*F* statistic	95% confidence interval	*p* value
Latencies	Target word identification accuracy (%)	−7.1856	*F*_(1,97)_ = 5.35	(−13.3505, −1.0207)	0.0228*
	Target word identification reaction times (seconds)	0.0283	*F*_(1,99)_ = 1.06	(−0.0261, 0.0828)	0.3050
	Comprehension question accuracy (%)	−9.3495	*F*_(1,99)_ = 4.73	(−17.8770, −0.8221)	**0**.0320*
	Subjective task load scores	9.7502	*F*_(1,99)_ = 19.28	(5.3446, 14.1558)	**<0**.0001*
Amplitudes	Target word identification accuracy (%)	15.2740	*F*_(1,96)_ = 1.33	(−11.0552, 41.6033)	0.2524
	Target word identification reaction times (seconds)	−0.3018	*F*_(1,98)_ = 7.09	(−0.5268, −0.0768)	**0**.0091*
	Comprehension question accuracy (%)	11.4097	*F*_(1,98)_ = 0.41	(−23.8556, 46.6750)	0.5223
	Subjective task load scores	−6.0637	*F*_(1,98)_ = 0.34	(−26.5690, 14.4416)	0.5587

Faster wave V peak latencies were associated with enhanced identification of the target words embedded within the stories, better comprehension question accuracy, and lower subjective listening effort. Larger amplitudes additionally related to faster target word identification reaction times. Any influential outliers observed for either the wave V condition effects (Fig. 3*B*) or behavioral metrics alone (Fig. 4*A*) were also excluded from these analyses. *p < 0.05.

## Discussion

We evaluated the effects of SiN and selective attention on low-level brainstem encoding. Our novel experimental paradigm permitted simultaneous measurement of both ABRs and behavior in both single- and dual-talker conditions using chirp-modified speech. ABRs recorded in the presence of a competing talker were associated with smaller amplitudes and delayed latencies compared with the mono-talker condition. We did not observe an effect of selective attention on our wave V measures. However, subcortical processing was related to multiple listening behavior metrics. Faster wave V latencies were associated with better accuracy of color word identification as well as higher comprehension question accuracy and lower scores of subjective listening effort. Meanwhile, more robust wave V amplitudes corresponded to faster reaction times for target word identification during the listening task. Collectively, the results suggest that faster, more robust neural encoding within the auditory brainstem may contribute to successful SiN listening processes.

The effect of auditory attention on subcortical responses has long been debated. For example, Forte et al. (2017) and Saiz-Alía and Reichenbach (2020) noted larger computationally derived auditory brainstem amplitudes for attended over unattended speech. Other studies have shown modulation of transient-evoked ABRs while attending to the auditory modality versus another (typically visual) domain (Lukas, 1980; Bauer and Bayles, 1990; Ikeda and Campbell, 2021, 2024; Kumar et al., 2023) or with increasing cognitive interference (Sörqvist et al., 2012; Brännström et al., 2020). Germane to our Cheech design, Strauss et al. (2025) recently provided evidence for a selective attention-based enhancement of wave V to chirps but not tone bursts. Subcortical attentional modulation has also been reported for sustained FFR (Hoormann et al., 2000; Galbraith et al., 2003; Hairston et al., 2013; Lehmann and Schönwiesner, 2014; cf. Varghese et al., 2015). Yet, the ABR has historically been viewed as largely immune from attention effects (Amadeo and Shagass, 1973; Picton and Hillyard, 1974; Connolly et al., 1989; Gregory et al., 1989). Our neural results were more strongly influenced by the presence of a competing talker than attended versus unattended conditions, suggesting a more limited capacity for attentional modulation of the ABR. These discrepancies across studies likely result from differences in experimental paradigms. It is possible that attention-related effects on subcortical processes are most effectively revealed when another attention-demanding task is presented concurrently with the auditory stimuli (Lukas, 1980; Brännström et al., 2020; Kumar et al., 2023), when the stimuli are more ecologically valid and relevant for real-world listening, as in the case of continuous speech (Ding and Simon, 2012; Zion Golumbic et al., 2013; Forte et al., 2017; Backer et al., 2019; Etard et al., 2019; Teoh and Lalor, 2019; Saiz-Alía and Reichenbach, 2020; Teoh et al., 2022; Carta et al., 2024; Shehabi et al., 2025), for stimuli presented at softer intensity levels (Schwent et al., 1976), or for more broadband chirp stimuli (Strauss et al., 2025). Future work should clarify the extent to which these different factors influence the detection of attention effects within the brainstem.

In our exploratory analyses, faster and/or larger Cheech-evoked wave V responses were associated with more successful speech-listening performance, including behavioral measures of speech identification (i.e., identification accuracy, RTs) as well as comprehension questions and subjective reports of listening effort. That is, more robust encoding in lower-level brainstem generators was related to higher-level speech recognition abilities, with and without the presence of a competing talker. Subcortical responses have been associated with other speech-listening behaviors including SiN perception (Papakonstantinou et al., 2011; Bramhall et al., 2015; Saiz-Alía et al., 2019; Grant et al., 2020; Mepani et al., 2020), self-reports of speech-listening abilities (Anderson et al., 2013), early categorization of speech phonemes (Carter and Bidelman, 2023; Rizzi and Bidelman, 2023), or even measures of auditory processing disorders (Omidvar et al., 2023). However, some studies have found no relationship between click-evoked ABR measures and speech perception deficits in noise (Grose et al., 2017; Smith et al., 2019; DiNino et al., 2025). It may be possible that our continuous, Cheech stimuli evoke subcortical neural processes more closely tied to meaningful behavioral outcomes. Collectively, our results provide further support for the important role of the brainstem in speech perception, both in quiet and the presence of a masking talker, as well as higher-level narrative comprehension and subjective perception of task-related listening effort.

As expected, the presence of a competing talker was associated with weaker brainstem encoding (i.e., wave V reduced amplitudes, delayed latencies), worse comprehension accuracy, and higher ratings of subjective task load. Compared with speech in a quiet background, the presence of a second talker introduced auditory masking (i.e., in both ears given the HRTF filtering), and the observed effects align well with previous work demonstrating delayed ABR wave V latencies and decreased amplitudes with ipsilateral masking (Burkard and Hecox, 1983; Verhulst et al., 2018; Saxena et al., 2022). Meanwhile, SiN perception (the “cocktail party problem”) is an important ability for listening in real-world environments, and some speech-hearing difficulties are only exposed when stimuli are presented in background noise. It remains possible that the variability of SiN perceptual skills, even among normal hearing listeners, could be traced to low-level neural encoding differences (Bharadwaj et al., 2015; Bramhall et al., 2015, 2017; Jain et al., 2019) or even top–down corticofugal modulations that aid neural processing despite the presence of masking noise (Price and Bidelman, 2021). Additionally, the degree to which the chirps and speech fuse as a unified perceptual object or “template,” particularly in the presence of a competing, masking signal, may further affect top–down attentional processes (Shinn-Cunningham, 2008) which, in turn, could influence how listeners perceive and process Cheech-in-noise. Varied contributions (or degeneration/selective loss) of auditory nerve fibers during speech encoding, particularly those with low-spontaneous discharge rates, could also help explain individual SiN differences (Mehraei et al., 2016, 2017). While the current study cannot differentiate between these theories—since both top–down and/or bottom–up mechanisms could alter ABR wave V and its relationship with speech perception—our results provide evidence of subcortical involvement for continuous SiN processing and suggest a possible neural factor for individual perception differences.

Contrasting with our findings, previous studies that compared male and female talkers reported larger wave V response amplitudes for male voices compared with female voices (Forte et al., 2017; Saiz-Alía and Reichenbach, 2020; Polonenko and Maddox, 2021). One explanation could be due to differences in stimulus design. Our Cheech and/or female voice modulation processes could have affected perceptual clarity and thus impacted brain and/or behavioral responses even though average RMS values were equated across the stories. Our female-sounding voice was modified from the original male voice (same talker for all stories) to maintain similar intonation, phrasing, and other expressive characteristics across the stories; only the speaker’s F0 and vocal tract length parameters were resynthesized using MATLAB STRAIGHT to create a female-sounding voice (Kawahara and Morise, 2011). The voice modulation may also have impacted the relative chirp-to-speech energy leading to differences across the male and female talkers which may have influenced specific cochlear activation patterns (and thus neural responses) in this study. Additionally, higher rates of stimulation are associated with reduced ABRs (Don et al., 1977; Weber and Fujikawa, 1977; Fowler and Noffsinger, 1983). Higher F0s and thus faster glottal pulse rates could explain subcortical encoding differences from speech stimuli spoken by different talkers (Polonenko and Maddox, 2021). While our stimuli had ∼10% more chirps for female-voiced stories compared with the male-voiced stories (i.e., faster glottal pulses creates more chirp-embedding opportunities), we set a minimum interstimulus interval of 18.2 ms between chirps to reduce overlapping activity and detrimental effects of high stimulation rates on neural encoding processes. We also equated the number of chirp trials across conditions in our analyses to avoid any systematic confounds produced by talker sex-based SNR differences due to imbalanced chirp events. Likely due to these factors, we did not observe characteristic male–female neural differences in our study (except those driven by the mono male compared with the dual-talker female condition). Finally, chirps are also designed to activate the entire cochlea in a near-synchronous fashion which produces more robust ABRs in general (Elberling et al., 2007, 2010) which could explain neurophysiologic differences between our study and other computationally derived methods more generally.

### Limitations

Our male and female talkers were spatially separated using HRTFs, and the talker locations were dynamically swapped with each other on average every 6 s to track spatially driven selective attention similar to Teoh and Lalor (2019). Pilot testing confirmed that the combined effects of pitch modulation (to produce a female-sounding voice) and spatial separation led to better listening performance, consistent with previous literature demonstrating talker sex, spatially separated release of masking effects in multitalker environments (Oh et al., 2021, 2022). However, gaze direction (as when participants were asked to follow the target cue across the two screens) as well as selective attention to lateralized sounds (including chirps) could potentially modulate neural or myogenic activity that contaminate responses (O’Beirne and Patuzzi, 1999; Patuzzi and O’Beirne, 1999; Bidelman et al., 2024; Mai et al., 2025). In our experiment, only male-spoken stories were included for the mono-talker condition. Female mono-talker stories were omitted from the final experiment after pilot testing to reduce the total testing time and potential fatigue. Participants therefore receive more exposure to the male voice during the speech-listening task. Thus, we cannot rule out certain idiosyncratic effects of our experimental paradigm on the observed brainstem–behavior relationships. Different experimental designs may lend better to other types of analyses, such as mediation analyses, that may shed more light on whether brainstem responses mediate the relationship between speech-listening conditions and behavioral outcomes. Future studies, both involving Cheech and other (ideally continuous) speech stimuli, may help elucidate the underlying neural encoding mechanisms involved in processing individual talker characteristics and their impact on speech perception abilities.

### Conclusion

Collectively, our results suggest a role, at least in part, for brainstem encoding process in individual speech perception abilities including SiN recognition, perceived listening effort, and comprehension. These findings reveal potential links between lower-level auditory structures and speech processing, even in audiometrically normal listeners, though future work is needed to further clarify the influence of competing talkers, selective attention, and talker sex on these processes. Furthermore, our results may have implications for improved speech understanding of other populations (e.g., older adults, children, individuals with hearing loss or assistive hearing devices, etc.) and certain speech processing disorders. Our study highlights the advantage of a stimulus like Cheech and more ecologically valid paradigms to uncover relationships between lower-level neural processing and higher-level listening skills in real-world auditory environments.

## Synthesis

Reviewing Editor: Anne Keitel, University of Dundee Decisions are customarily a result of the Reviewing Editor and the peer reviewers coming together and discussing their recommendations until a consensus is reached. When revisions are invited, a fact-based synthesis statement explaining their decision and outlining what is needed to prepare a revision will be listed below. The following reviewer(s) agreed to reveal their identity: Lorenz Fiedler. Note: If this manuscript was transferred from JNeurosci and a decision was made to accept the manuscript without peer review, a synthesis may not be available.

Synthesis:

The manuscript has been reviewed by one original reviewer and one new reviewer. Both reviewers agreed that the study shows a valuable contribution to methodological innovation, and potentially to auditory brain stem processing of speech. Both reviewers also highlighted that the authors responded comprehensively to previous reviewer comments. Nonetheless, several new points came up that should be addressed in a major revision. You'll find a summary of both reviewers' comments below. Please respond to each comment in a point-by-point manner.

Overall assessment

There are some weaknesses that limit the interpretability of the findings. Beyond the issues already addressed and the additional points raised below, the study introduces a promising methodological contribution to auditory attention research. Specifically, the CHEECH paradigm integrates chirp-based stimulation-recently shown to be effective for probing auditory attention along the auditory pathway-within an ecologically valid framework using modified speech signals.

Comments regarding how previous points were addressed:

The manuscript has already undergone a successful peer-review process, receiving thoughtful and well-substantiated feedback from two reviewers. The authors have responded comprehensively to these comments in their rebuttal letter. Several of the reviewers' concerns were valid and, as acknowledged in the revised discussion, substantially diminished the interpretive weight of the study's findings.

The authors have done an extensive revision. The authors addressed most of the concerns and for the ones they could not address, they found reasonable arguments. They changed/added figures and thus increased the transparency of the results.

Conceptual points/framing/interpretation:

1. It is known that SiQ and signal-in-noise provide different latencies and amplitudes. The one-talker experiment is therefore quite confusing, particularly in the context of a pooled analysis. It is well established that lower stimulation intensity leads to reduced wave V amplitudes and increased latencies. This translates into a decreased signal-to-noise ratio (SNR) in the stimuli (e.g., Saxena et al., 2022 and references therein or modeling studies, e.g., Verhulst 2018). Specifically, ipsilateral noise tends to reduce wave V amplitudes and prolong latencies (unlike ipsilateral stimulation with contralateral noise masking in isolated conditions, which has a different effect). Given that the authors employed the SADIE II database and virtual sound sources (VSS) by HRTFs, the dual-talker condition effectively consists of signal plus noise, where the 'noise' corresponds to VSS energy from the competing talker. If this is the case, several latency and amplitude differences between the mono-talker (SiQ) and dual-talker (speech-in-noise) conditions may be attributable to the well-documented relationship between noise, amplitude, and latency in wave V responses. Please ensure that the findings regarding the one-talker condition are not overstated.

2. The authors did a good job addressing the reviewers' concerns in the discussion and additional analysis. However, this is not yet reflected in the overall framing of the results - particularly in the title and conclusions. A less assertive formulation might be more appropriate, such as 'Correlates of selective attention, comprehension, and subjective listening effort in auditory brainstem responses to speech in noise', or a similar phrasing that better aligns with the updated findings (e.g., compare line 592-594 to the title and conclusion). The study makes a valuable methodological contribution by translating the concept of using chirps in selective attention paradigms as recently suggested into ecologically valid listening environments. As mentioned, Point 3 might be an avenue to improve the method in future and Point 4 might help get more specific results from the already available data.

Methodological points

3. In Strauss et al. (2025), the authors employed full-range potentials to chirp stimuli in a dichotic selective auditory attention experiment using the classic Hillyard ERP paradigm (Hillyard et al. (1973)). The chirps were designed to balance synchronous cochlear activation within tonality (to get a streaming experience in the (pure) tone-bursts vs. chirp stimulation. The authors demonstrated robust effects of selective auditory attention on wave V of the auditory brainstem response (ABR) under chirp stimulation, most clearly reflected in the spectrotemporal consistency of single trials-specifically, the inter-trial phase coherence (ITPC) of the auditory response recordings (ARR) within a defined frequency band. These findings provide compelling evidence for the utility of chirp stimuli in attention paradigms. While Strauss et al. (2025) employed a traditional ERP framework, the authors of the present study adopted a more ecologically valid design by embedding chirps within speech. Both approaches converge on the notion that chirps can enhance brainstem encoding of attended signals-effectively giving 'more punch' to the early auditory pathway. Importantly, the results of Strauss et al. (2025) suggest that a readily matchable auditory 'template' (in the sense of the biased competition theory (Desimone &Duncan, 1992), as adapted to the auditory domain (Shinn-Cunningham, 2008)) may be necessary to engage corticofugal projections to the inferior colliculus. The CHEECH stimuli used by the authors appear well-suited to this purpose, provided that speech and chirps fuse into a coherent auditory object in very early processing stages of the hearing path (a perceptual object or cortically fused object does not imply a consistent tonotopical representation at earlier processing stages, e.g., see Pantev et al. (1996), and might thus not be targeted as such by attention-driven top-down projections). While this hypothesis is difficult to prove or falsify directly, it warrants further discussion. For instance, could the Cheech method be refined to better promote early object fusion and corticofugal engagement? Please also acknowledge that chirps have already shown potential for revealing clear ABR attention effects in classic ERP paradigms.

4. A very sensitive and transparent analysis for ABRs is the spectrotemporal consistency in terms of a inter-trial phase coherence (see Corona-Strauss et al., 2009), particularly with regard to selective attention correlates in the ABR (see Strauss et al., 2025). As the authors refer to 'borderline statistics' for the relevant contrasts (see Comments of R1 in the first round and also COMMENT 1), such a more sensitive analysis could be very helpful in identifying where the discriminant energy between target and masker resides in time and frequency. This is especially important given the proximity of time-frequency energy clusters elicited by chirp stimuli (see Strauss et al., 2025); in this context, resolution in both time and frequency becomes a critical factor. For instance, the non-causal 6th-order Butterworth filter used by the authors at a sampling rate of 8192 Hz already spans a substantial portion of the ABR range. Since the authors employed an additional mastoid electrode, the standard Cz-mastoid configuration might suffice (see the comparison in Strauss et al. (2025) between the 128-channel setup and the classic Cz-mastoid configuration). This could significantly simplify the otherwise complex preprocessing pipeline, at least for the attention effects reflected in ABRs. We also highly recommend sharing the data and code.

5. It would be very helpful if the authors could provide additional information about the chirp generation process. One reviewer followed the reference to the 'established protocol' cited in their 2019 paper, but this ultimately led to a patent citation, which made the trail somewhat cumbersome. While we understand that only a few publications address frequency-(band)-specific chirps in detail, the design method is particularly relevant for the CHEECH approach presented here-especially in light of the previously discussed considerations (e.g., tonality, integration into the speech signal, pitch differences between male and female voices, and the corresponding need for differential compensation of basilar membrane traveling time). Since this may be of interest to readers concerned with selective attention to auditory objects within a stream, a brief explanation-perhaps in the supplementary material-would be greatly appreciated. It would also be important to mention that embedding frequency-(band)-specific chirps into speech activates only the corresponding proportion of the cochlea. This might offer an additional explanation for why the observed contrasts are smaller than those reported in Strauss et al. (2025), who used tonal broadband chirps.

6. The authors use rather loud stimulation and base their arguments on ecological validity. However, at least for later potentials, the attention effects are better visible with soft stimulation (e.g., see Hillyard et al. 1995). Also in Strauss et al. 2025, a markedly softer stimulation was used to (chirp-)evoke selective attention at the brainstem. This can be tested in future work but is probably worth mentioning in the discussion, as the CHEECH method might provide more contrast in those regimes. The good news about chirps that they provide robust ABRs for rather soft stimulation levels (Dau et al. 2000, Corona-Strauss et al. 2009).

Minor comments:

- Streamlining the writing in the Methods section would be helpful. The authors report only a subset of their data, which is perfectly acceptable. However, unfamiliar readers are likely to examine every aspect in detail-and only later discover that certain data or procedures will be presented in a separate report, or that it's unclear which elements belong to which part of the study (e.g., see lines 310-313 and the preceding text). Similarly, lines 326-327 mention "clicks for separate recordings" - but what recordings? Are they relevant to the current study or reserved for future reports? Of course, all elements must be reported for reasons such as duration, sequence effects, etc., but this should be done as early and as concisely as possible in the manuscript to increase readability and avoid confusion.

- Please replace the infinity in line 217 by a value (should be fs/2 of audio sampling rate?).

- In lines 778-780 the authors refer to gaze as a source of myogenic activity from the auricular muscles that could potentially contaminate the responses; note that spatial auditory attention also modulates these auricular muscles to speech and chirps; e.g., see Mai et al. (2025) for chirp tones as stimuli.

References:

Verhulst S, Altoè A, Vasilkov V. Computational modeling of the human auditory periphery: Auditory-nerve responses, evoked potentials and hearing loss. Hear Res. 2018; 360:55-75

Saxena U, Shukla B, Tripathy R. Impact of Noise on Sound Processing at Lower Auditory System: An Electrophysiological Study. Indian J Otolaryngol Head Neck Surg. 2022 Dec;74(Suppl 3):4131-4137.

Strauss DJ, Corona-Strauss FI, Mai A, Hillyard SA, Unraveling the effects of selective auditory attention in ERPs: From brainstem to cortex. Neuroimage 2025, 316:121295

(an earlier preprint from Feb 2024 was published as "Fifty Years After: The N1 Effect Travels Down to the Brainstem" at bioRxiv, https://doi.org/10.1101/2024.02.23.581747)

Shinn-Cunningham BG. Object-based auditory and visual attention. Trends in Cognitive Sciences;12(5):182-186.

Hillyard SA, Hink RF, Schwent VL, Picton TW. Electrical signs of selective attention in the human brain. Science. 1973;182(4108):177-180.

Hillyard SA, Münte TF. Selective attention to auditory stimuli. In: Gazzaniga MS, editor. The Cognitive Neurosciences. Cambridge, MA: MIT Press; 1995.

Dau T, Wegner O, Mellert V, Kollmeier B. Auditory brainstem responses with optimized chirp signals compensating basilar-membrane dispersion. J Acoust Soc Am. 2000

Corona Strauss FI, Delb W, Schick B, Strauss DJ. Phase stability analysis of chirp evoked auditory brainstem responses by Gabor frame operators. IEEE Trans Neural Syst Rehabil Eng. 2009; 17(6):530-536

Fobel O, Dau T. Searching for the optimal stimulus eliciting auditory brainstem responses in humans. J Acoust Soc Am. 2004;116(4 Pt 1):2213-2222

Pantev C, Elbert T, Ross B, Eulitz C, Terhardt E. Binaural fusion and the representation of virtual pitch in the human auditory cortex. Hearing Res. 1996

Mai, A, Hillyard, SA., Strauss, DJ., Corona-Strauss, FI. Selective Listening to Unpredictable Sound Sequences Increases Tonic Muscle Activity in the Human Vestigial Auriculomotor System. eNeuro, 2025, doi: 0.1523/ENEURO.0301-25.2025
