# Supplementary material for: Auditory Brainstem Encoding of Speech-in-Noise Predicts Word Identification, Narrative Comprehension, and Subjective Listening Effort in a Selective Attention Task
Source: eNeuro. 2026 Sep 4;13(9):ENEURO.0272-25.2026. doi: 10.1523/ENEURO.0272-25.2026 (PMC13629154; doi:10.1523/ENEURO.0272-25.2026)
Supplement: Supplementary file 1 [file eneuro-13-ENEURO.0272-25.2026-s001.docx]

# Extended Data Figure 1-1: Short story stimulus details.

The stories used in this study were selected for content unlikely to be widely known, to avoid comprehension question recall based on modern renditions (i.e., popularized by children’s books or movies), and with story arc progress within a 7.5-minute block (i.e., rising action, climax, or falling action) to ensure sufficient and generally consistent content for probing comprehension. Thirteen stories were piloted on four raters who scored them according to factors such as interest and engagement levels, ease of understanding, grammar or writing style complexity, familiarity (i.e., low familiarity preferred), and suitability for an adult audience. Seven stories that scored highest across these factors were ultimately retained for the experiment. The seven stories were then divided into separate experimental conditions: two stories presented alone (“mono-talker”), two sets of paired stories (“dual-talker”), and one for initial practice. Given the constraint of naturalistic color word embeddings within each story, color words occasionally overlapped within the dual-talker pairings. The story pairs were determined according to those which had the fewest color word overlaps, and any overlapping color words were dropped from the behavioral task analyses to distinguish target story hits from masker story false alarms. As mentioned in the manuscript, three stories—one mono-talker story and a pair of dual-talker stories—were further modified during the Cheech process as part of a separate study. We do not include details or analyses of those stories here. The table below describes details for the remaining three experimental stories (i.e., one mono-talker story and a pair of dual-talker stories) plus the story used for practice. Story block presentation order was pseudo-randomized to ensure the same dual-talker story pairs were not heard back-to-back, and the order of all the blocks was counterbalanced across participants to reduce influence of any learning effects.

| **Story title** | **Author** | **Collection** | **Experimental condition** | **Male or female voice** |
| --- | --- | --- | --- | --- |
| The Master Cat, or Puss in Boots | Charles Perrault, translated by J. E. Mansion and Robert Samber | The Fairy Tales of Charles Perrault | Mono-talker | Male |
| Lily and the Lion | Jacob Grimm & Wilhelm Grimm | Grimms’ Fairy Tales | Dual-talker | Male |
| The Four Clever Brothers | Jacob Grimm & Wilhelm Grimm | Grimms’ Fairy Tales | Dual-talker | Female |
| The Wanderers | Edith Howes | Maoriland Fairy Tales | Practice only (mono-talker) | Male |
