# Supplementary material for: Auditory Brainstem Encoding of Speech-in-Noise Predicts Word Identification, Narrative Comprehension, and Subjective Listening Effort in a Selective Attention Task
Source: eNeuro. 2026 Sep 4;13(9):ENEURO.0272-25.2026. doi: 10.1523/ENEURO.0272-25.2026 (PMC13629154; doi:10.1523/ENEURO.0272-25.2026)
Supplement: Supplementary file 2 [file eneuro-13-ENEURO.0272-25.2026-s002.docx]

# Extended Data Figure 1-2: Cheech synthesis parameters.

As mentioned in the Stimuli section, one voice within the dual-talker story pair was pitch shifted prior to the Cheech modification process to simulate a female speaker. Consistent with combined voice-sex and spatial release from masking outcomes observed in previous work (Oh et al., 2021, 2022), two distinct talker F0s helped listeners better distinguish between the spatially-separated voices and more successfully complete the task during piloting. MATLAB STRAIGHT parameters associated with the female voice manipulation are listed below. Initially, female mono-talker stories were planned for the full experiment as well (i.e., resulting in nine stories instead of seven), but it became clear during piloting that additional stories would significantly increase the total testing time and lead to excessive fatigue, so these stories were ultimately dropped from the final experimental design.

| **Cheech Parameter** | **Value** |
| --- | --- |
| Fs (sample rate; Hz) | 48000 |
| Speech filter bands (Hz) | 0-250; 500-1000; 2000-4000; 11000+ |
| Chirp filter bands (Hz) | 250-500; 1000-2000; 4000-11000 |
| Speech filter order | 10 |
| Chirp filter order | 4 |
| Voicing (speech envelope) filter, to identify voicing by power (Hz) | 20-1000 |
| Voicing threshold, for identifying sufficient glottal pulse energy (normalized amplitude) | 0.03 |
| Minimum voiced period to chirp (sec) | 0.05 |
| Minimum chirp ISI (sec) | 0.0182 |
| Minimum gap after first chirp (sec) | 0.048 |
| Maximum voicing duration, above which gaps were inserted (intra-voicing gap) to prevent very long chirp trains especially during loud speech modulations (sec) | 0.4 |
| Intra-voicing gap (sec) | 0.05 |
| Normalized chirp amplitude | 0.10 |
| Female voice characteristics  Resynthesized voice mean F0 pitch (Hz)  Maximum frequency modulation (Hz)  Vocal tract length warping factor | 180 4000  1.04 |
| Resynthesized voice characteristics   Score, to reduce thumping in the resynthesized audio  Margin, to reduce buzzing in the resynthesized audio | 0.6  0.6 |
